# Schizencephaly Associated With Bipolar Affective Disorder

**DOI:** 10.7759/cureus.54534

**Published:** 2024-02-20

**Authors:** Rahul Rama Rao, Anish Bandalore Satheesha Reddy, Dhanushia P, Abhay Koul

**Affiliations:** 1 Psychiatry, Mysore Medical College and Research Institute, Mysuru, IND; 2 Psychosis Studies, King’s College London, London, GBR

**Keywords:** neurodevelopmental, congenital, mania, bpad, psychosis, bipolar, schizencephaly

## Abstract

Schizencephaly is a rare congenital anomaly characterized by the formation of abnormal clefts in the brain. Despite the association of psychotic symptoms with various brain abnormalities or insults, their occurrence in individuals with schizencephaly is relatively infrequent. The association of bipolar disorder, with or without psychosis, with schizencephaly is rarer. A systematic search on PubMed using "Schizencephaly AND Bipolar Disorder" yielded only four case studies specifically addressing the connection between these two conditions. Here, we present a case of a 22-year-old male patient with a history of childhood seizures who developed first episode psychosis along with manic symptoms and was found to have closed-lip schizencephaly.

## Introduction

Schizencephaly is a rare congenital neuronal migration disorder of the brain characterized by clefts or fissures lined by heterotrophic gray matter, which connects the surface of the cerebral hemispheres with the lateral ventricle [1]. Two main types of malformation depending on its communication with the ventricular system have been distinguished: closed-lip and open-lip schizencephaly [2]. Schizencephaly can be either unilateral or bilateral and has a prevalence estimated to be 1.48 per 100000 live births [3].

While the underlying causes of schizencephaly remain poorly understood, studies suggest that the condition has a multifactorial etiology involving genetic, environmental, and developmental factors. Mutations in several genes have been associated with the development of schizencephaly, including the EMX2 gene [4], which is involved in the regulation of brain development. Environmental factors, such as maternal infections, exposure to toxins, and other prenatal factors, have also been associated with schizencephaly [5].

Schizencephaly has been linked to various neurodevelopmental disorders, including intellectual disability, epilepsy, cerebral palsy, and motor deficits [6]. Recent research has indicated a potential association between schizencephaly and psychiatric disorders, with further investigations specifically exploring the relationship between schizencephaly and bipolar disorder [7,8]. These findings contribute additional evidence to the growing understanding that disrupted neurodevelopmental pathways associated with schizencephaly may play a role in the underlying mechanisms of bipolar disorder and other psychiatric conditions. This report aims to enhance the current literature by raising awareness regarding the potential association between schizencephaly and bipolar I disorder.

## Case presentation

A 22-year-old male, right-handed, educated till primary school, employed as a laborer, with a well-adjusted pre-morbid personality, was brought by his mother to the psychiatric outpatient department. His presenting complaints included excessive talkativeness, reduced sleep, heightened irritability, and extravagant claims. He expressed a strong desire to pursue higher education, as he envisioned himself to be a professor in an educational institution. The patient also displayed grandiose delusions, which became apparent when he firmly believed and identified himself as a police officer.

According to the patient’s mother, these behavioral changes began 20 days before the visit to the department. She claimed he had recently become irritable and lost his temper when confronted about it. She also noticed he was excessively preoccupied with religious matters, praying at odd hours of the day, which was unusual for him.

The mother reported that he had experienced seizures during his childhood, but she could not recall the specific episodes or whether any tests or medications had been prescribed for them. There was no family history of any psychiatric disorders; however, there was an intellectual disability in a first-degree relative. The antenatal, natal, and postnatal periods were uneventful, and there was no history of developmental delays.

During the neurological examination, no specific focal neurological deficits were identified. On mental status examination, the patient was unkempt and exhibited psychomotor agitation with difficulty establishing rapport. The patient's speech was spontaneous, relevant, and coherent, but notable alterations were observed, including increased tone, tempo, volume, and decreased reaction time. Subjectively, the patient reported normal mood, but objectively, elation with intermittent episodes of irritability and emotional lability was noted.

The patient did not exhibit any perceptual abnormalities during the examination. However, there were notable deficits in attention, with a tendency for arousal but an inability to sustain focus. Additionally, the patient displayed impaired social judgment and a lack of insight into their condition or behavior. The patient's symptoms and examination findings suggested he was experiencing a manic episode with psychotic symptoms. The patient was diagnosed with Bipolar I disorder as per the International Classification of Diseases (ICD), 11th revision criteria.

**Figure 1:**
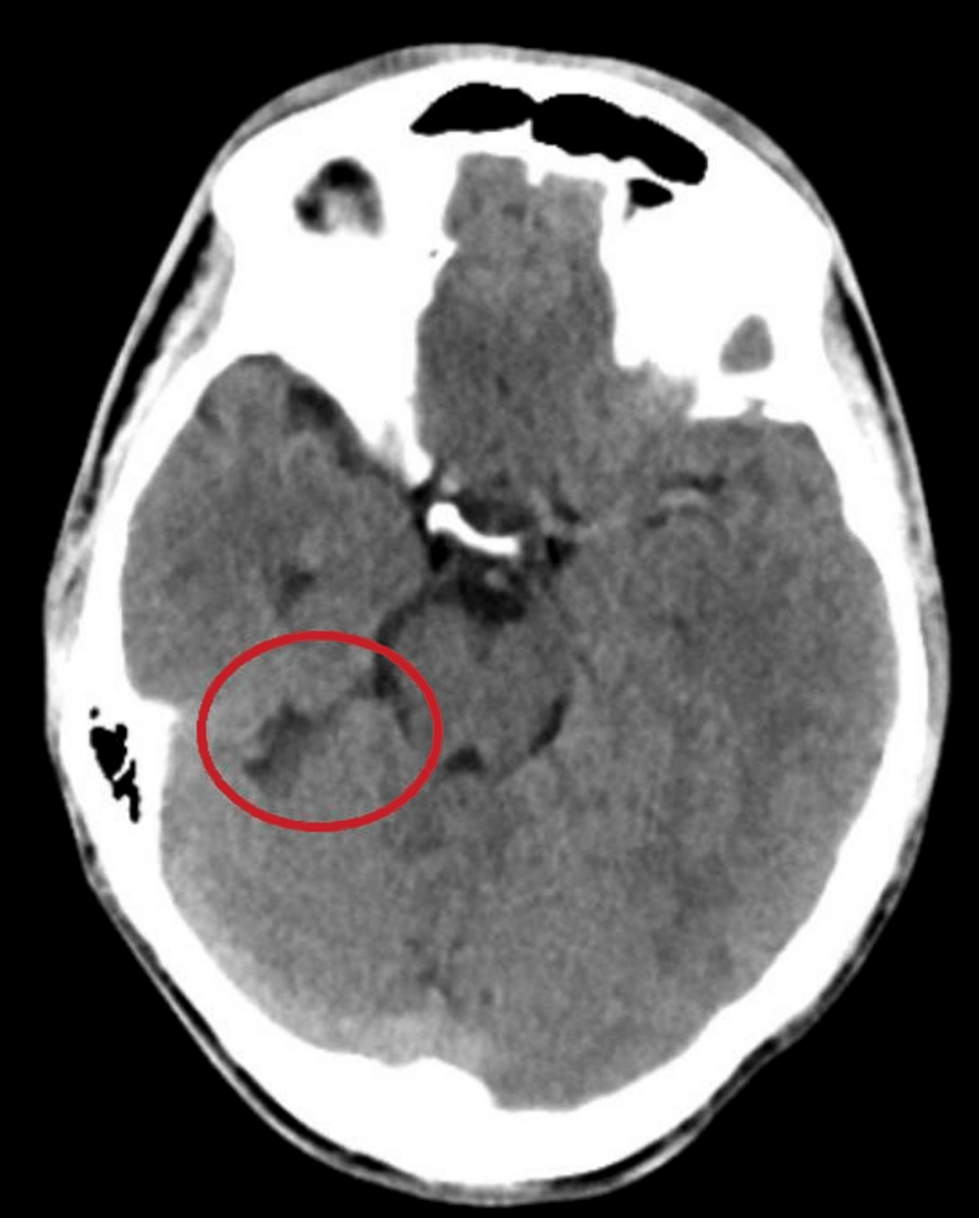
CT head without contrast demonstrating right temporal lobe closed-lip schizencephaly

During his hospital stay, all routine blood investigations were ordered. It was found that his differential neutrophil count was elevated, prompting a blood culture, which yielded unremarkable results. An electroencephalogram (EEG) revealed no abnormalities. CT brain revealed the presence of a cerebrospinal fluid-filled cleft in the right temporal lobe, which demonstrated communication with the ambient cistern. The walls of the cleft displayed close apposition to each other. No obvious gliosis or volume loss was noted. No significant abnormalities were observed in the brain parenchyma, cortical sulci, cisterns, or ventricular system (Figure 1). These findings were consistent with a diagnosis of right temporal closed-lip schizencephaly.

Before starting treatment, the severity of manic symptoms at baseline was assessed using the Young Mania Rating Scale (YMRS), which revealed a score of 30. The patient was initiated on a treatment regimen of sodium valproate 1000 mg/day (administered as two equally divided doses of 500 mg), olanzapine 10 mg/day, and clonazepam 0.5 mg/day, given at night. After two weeks, the patient exhibited significant improvement and was discharged.

The patient attended a follow-up appointment after 20 days, during which the YMRS was reassessed, revealing a score of 15. Notably, the patient had achieved pre-morbid levels of functioning and did not exhibit any depressive symptoms or suicidal ideation. It was recommended that the patient continue with the current medication dosage, and follow-up appointments were scheduled at six weeks and three months.

## Discussion

We have presented a noteworthy case involving a close-lip right temporal schizencephaly in a patient who presented with first-episode psychosis and subsequently received a diagnosis of Bipolar I disorder. Through a review of existing literature, it was found that only four cases with similar associations have been previously reported [7,9-11]. Therefore, this case report serves as a valuable addition to the body of knowledge regarding the psychotic manifestations of schizencephaly.

The neurodevelopmental model proposes that disrupted or deviant brain development during childhood, as seen in conditions such as intellectual disability, autism spectrum disorder, and attention deficit hyperactivity disorder, as well as in adult psychiatric disorders like bipolar disorder and schizophrenia, may contribute to their etiology [6]. Organic brain anomalies, such as schizencephaly, usually diagnosed through magnetic resonance imaging (MRI) and computed tomography (CT) scans, can disrupt the process of neuronal migration and neurogenesis, potentially affecting specific neuronal pathways. It has been postulated that these aberrant neuronal pathways may play a role in the emergence of psychotic manifestations later in an individual's life [12]. Schizencephaly can thus be considered as an initial factor in the two-hit hypothesis model for psychiatric disorders such as schizophrenia and bipolar affective disorder [13].

The presented case included a childhood history of seizures with no reported recent episodes, which prompted us to perform a neuroimaging workup. ​​While our decision was tailored to the specifics of our case, an incidental finding of schizencephaly reiterates the role of neuroimaging in first-episode psychosis [14]. Further, the presence of temporal lobe schizencephaly raises the possibility of a potential association with epilepsy in the patient.

Through our review of case reports, we noted that symptoms showed improvement with the use of second-generation antipsychotics [7,9]. In this case, we observed a positive treatment response using a combination of sodium valproate and clonazepam, along with a second-generation antipsychotic, olanzapine. This reaffirms the efficacy of recommended treatment protocols for psychiatric disorders in individuals with pre-existing organic anomalies [15].

## Conclusions

Schizencephaly, a neurodevelopmental disorder, rarely presents with a psychotic phenotype. In light of the patient's history of childhood seizures, we conducted EEG and brain imaging examinations, leading to the incidental discovery of schizencephaly. Therefore, it can be inferred that including a diagnosis of schizencephaly in the list of possible differential diagnoses could be beneficial in cases of early-onset psychosis accompanied by motor or intellectual deficits, with or without a history of seizures. While further research is imperative to comprehensively elucidate the intricate relationship between these factors, the neurodevelopmental model provides a valuable framework for understanding the underlying mechanisms of these disorders and lays the groundwork for developing novel treatment strategies.
